# Supplementary material for: Anticancer Effects of BRD4 Inhibitor in Epithelial Ovarian Cancer
Source: Cancers (Basel). 2024 Feb 27;16(5):959. doi: 10.3390/cancers16050959 (PMC10930785; doi:10.3390/cancers16050959)
Supplement: Supplementary file 1 [file cancers-16-00959-s001.zip › cancers-2848783-supplementary.pdf]

cancers

MDPI

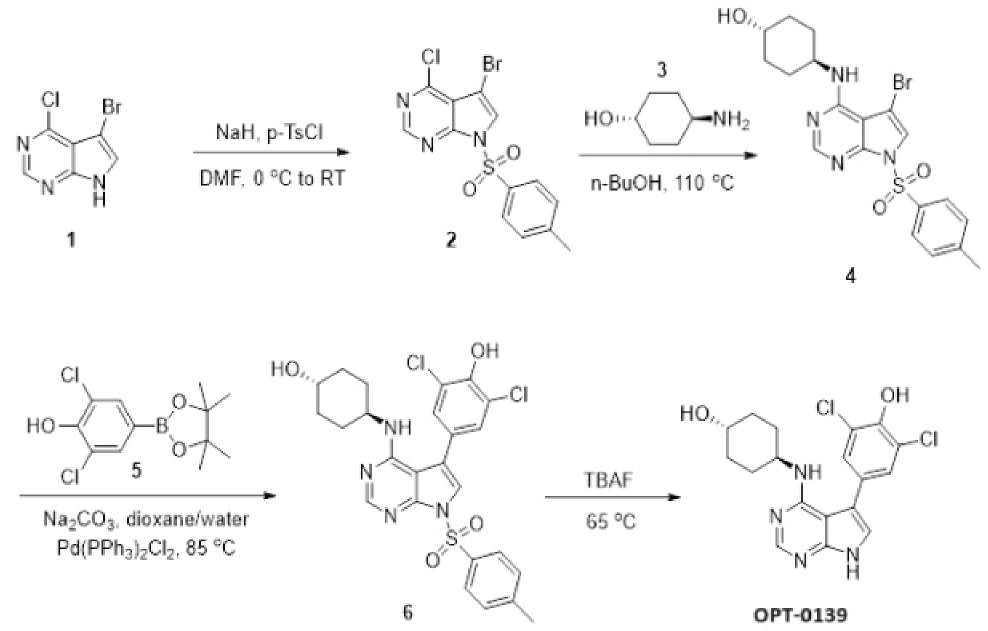

Figure S1. Molecular Formula of OPT-0139.

Table S1. Compilation of PCR Primers Employed in Experimental Procedures.

Gene	Forward	Reverse
BRD4	CTTTCTGCACCAGTGGAGACTT	GGACTGTTAGTTAGAACTGCAGGA
p21	ACTTTGTCACCGAGACACCAC	GTAGAAATCTGTCATGCTGGTCTG
p27	CTTGCCCGAGTTCTACTACAGAC	GAACCGTCTGAAACATTTTCTTCT
c-Myc	CCCCCAAGGTAGTTATCCTTAAAA	TTGAGGCAGTTTACATTATGGCTA
Hif-1a	ACCTACTGCAGGGTGAAGAATTAC	CAATGCAATGGTTTAAATACCAAA
Vegf	TGAATGCAGACCAAAGAAAGA-TAG	TCTGCAAGTACGTTCGTTTAACTC
Nanog	TCCCTTCTATAACTGTGGAGAGGA	GGTTGCATGTTCATGGAGTAGTTT
Oct-4	ACATCAAAGCTCTGCAGAAA-GAAC	TTTTCATTGTTGTCAGCTTCCTCC
Bcl-2	ATGCCTTTGTGGAACTGTACG	ATTGTTGTGTGTGTGTGTGTCTGT
Bax	GAGTCACTGAAGCGACTGATGT	AGGAGGTTTATTACCCCCTCAAG
